# Cannabis use in first episode psychosis in Tunisia

**DOI:** 10.1192/j.eurpsy.2024.630

**Published:** 2024-08-27

**Authors:** E. Bergaoui, R. Lansari, O. Chehaider, W. Ben Flah, A. Larnaout, W. Melki

**Affiliations:** Psychiatry D, Razi hospital, Manouba, Tunisia

## Abstract

**Introduction:**

The use of cannabis is associated with developing psychotic disorders, especially for those with a pre-existing vulnerability and elevated familial risk for psychosis.

**Objectives:**

To assess cannabis use during first episode psychosis and its relationship with patients’ clinical symptoms and functioning.

**Methods:**

We assessed 50 patients hospitalized for first episode psychosis using three scales: CAST test (Cannabis Abuse Screening Test), Positive and Negative Syndrome Scale (PANSS) and Global Assessment of Functioning (GAF).

**Results:**

The sex ratio of our population was 4 men to 1 woman. The average age was 25.6±6.16 years. About 60% of the patients used cannabis. The average duration of untreated psychosis was 10 months, with extremes ranging from one week to 24 months. Forty-four patients were antipsychotic-naïve (88%). For patients who used cannabis, the mean score of CAST test was 11.3±4.16, with extremes between 4 and 18. The risk of dependence was high in 81% of cannabis users. The PANSS total scale showed a mean score of 58.29±12.90 with extremes between 35 and 91. The average score at GAF scale was 30 with extremes between 20 and 70. Duration of untreated psychosis was significantly correlated to negative scale of PANSS (p=0,012 ; r=0,420), PANSS total score (p=0,011 ; r=0,424) and GAF levels (p=0,012 ; r=-0,420).

There was no association between age of onset of psychosis and cannabis use (p=0,181) nor CAST scores (p=0,747). There was no correlation between CAST and GAF scores (p=0,641).

However, there was a significant and positive correlation between CAST scores and positive scale of PANSS (p=0,04 ; r=0,432).

**Conclusions:**

Cannabis use is neither necessary nor sufficient to cause psychosis on its own. However, it has an influence on the prognosis. Early intervention programs should address cannabis and substance use problems early in the course of illness.

**Disclosure of Interest:**

None Declared

